# VP8, the Major Tegument Protein of Bovine Herpesvirus-1, Is Partially Packaged during Early Tegument Formation in a VP22-Dependent Manner

**DOI:** 10.3390/v13091854

**Published:** 2021-09-17

**Authors:** Soumya Sucharita, Kuan Zhang, Sylvia van Drunen Littel-van den Hurk

**Affiliations:** 1 Vaccine and Infectious Disease Organization, University of Saskatchewan, Saskatoon, SK S7N 5E3, Canada; soumya.sucharita@usask.ca; 2 Department of Biochemistry, Microbiology and Immunology, University of Saskatchewan, Saskatoon, SK S7N 5E5, Canada; 3 Department of Virology and Immunology, Shanghai Virogin Biotechnology Co. Ltd., Shanghai 201108, China; zhangkuan.100@163.com

**Keywords:** BoHV-1, major tegument protein, early tegument, tegument formation, virus maturation

## Abstract

Bovine herpesvirus-1 (BoHV-1) is a major cause of rhinotracheitis and vulvovaginitis in cattle. VP8, the major tegument protein of BoHV-1, is essential for viral replication in the host. VP8 is phosphorylated by the viral kinase US3, mediating its translocation to the cytoplasm. VP8 remains nuclear when not phosphorylated. Interestingly, VP8 has a significant presence in mature BoHV-1YmVP8, in which the VP8 phosphorylation sites are mutated. This suggests that VP8 might be packaged during primary envelopment of BoHV-1. This was investigated by mass spectrometry and Western blotting, which showed VP8, as well as VP22, to be constituents of the primary enveloped virions. VP8 and VP22 were shown to interact via co-immunoprecipitation experiments, in both BoHV-1-infected and VP8-transfected cells. VP8 and VP22 also co-localised with one another and with nuclear lamin-associated protein 2 in BoHV-1-infected cells, suggesting an interaction between VP8 and VP22 in the perinuclear region. In cells infected with VP22-deleted BoHV-1 (BoHV-1ΔUL49), VP8 was absent from the primary enveloped virions, implying that VP22 might be critical for the early packaging of VP8. In conclusion, a novel VP22-dependent mechanism for packaging of VP8 was identified, which may be responsible for a significant amount of VP8 in the viral particle.

## 1. Introduction

Bovine herpesvirus-1 (BoHV-1) belongs to the family *Alphaherpesviridae* and is a major cause of bovine rhinotracheitis and vulvovaginitis [1]; it also causes reduced milk production as well as infertility in cows and, hence, adversely affects the dairy industry [2]. The structure of BoHV-1 includes a proteinaceous 125 nm diameter capsid, which packages the double-stranded DNA of about 135 kb; the capsid is covered by a tegument, which consists of approximately 20 proteins [3]. A lipid envelope forms the outermost shell of the virus, and contains glycoproteins [4]. Tegument proteins are responsible for diverse functions, and around 11 tegument proteins are known to be conserved across alphaherpesviruses along with their interactions [5,6]. The life cycle of human herpesvirus-1 (HHV-1) and BoHV-1 starts by entry into the host cell via receptor-mediated endocytosis, which involves fusion of the viral envelope and the endocytic membrane due to interaction between the host cell molecules and glycoproteins [7,8]. 

During entry into the host cell, HHV-1 and BoHV-1 lose the lipid envelope, and also shed some of their tegument proteins. The capsid then releases the viral DNA into the nucleus through the nuclear pore complex, where the DNA replicates via a rolling circle mechanism [8,9]. The newly formed DNA is packaged into the capsid inside the nucleus, which is then ready to exit the nucleus via a budding process [8,10]. During egress, the capsid gains a primary envelope from the inner nuclear membrane, and buds into the perinuclear space [8,11]. It is known for HHV-1 that a few tegument proteins are also packaged at this stage of primary envelopment [12]. Subsequently, the primary enveloped virus particle fuses with the outer nuclear membrane and loses the primary envelope, while retaining some of the tegument proteins, and buds out of the nucleus [13]. As the virus particle proceeds through the cytoplasm towards its site of maturation at the Golgi, the tegument undergoes changes in its composition due to loss and/or addition of proteins [12]. At the Golgi, the final maturation of the virus takes place, after the addition of the lipid envelope and the glycoproteins [11].

VP8, the major tegument protein of BoHV-1, is a product of the *U_L_47* gene [14], and plays a versatile role in viral replication [4], induction of humoral and cell-mediated immunity [15], alteration of host defense mechanisms [16], and cell death or apoptosis [17]. It is a late protein, and is conserved throughout the alphaherpesvirus family [14]. The requirement for VP8 during BoHV-1 replication has been inferred from a 1000-fold reduction in viral titre in cells, and the total inability of a VP8-deleted BoHV-1 (BoHV-1ΔUL47) to replicate in cattle upon infection. In addition, the amounts of glycoprotein D (gD) and tegument protein VP22 were found to be significantly reduced in the mature BoHV-1ΔUL47 [4]. Early during infection with HHV-1 and BoHV-1, the *UL47*-encoded protein localises to the nucleus [18,19], which is mediated by nuclear localisation signals (NLSs). At later stages of infection, BoHV-1 VP8 and HHV-1 VP13/14 are exported, which is consistent with the presence of nuclear export signals (NESs) [19,20]. VP8 undergoes phosphorylation in the cytoplasm by host casein kinase 2 (CK2), and in the nucleus by a viral kinase—the unique short protein 3 (US3). This phosphorylation aids in many cellular functions of VP8, including host defense modulation and export of VP8 from the nucleus to the cytoplasm. When a mutant (BoHV-1YmVP8), in which the phosphorylation sites were replaced by alanine, was studied, it was found that VP8 remained nuclear [21], and in the mature BoHV-1YmVP8 virus particles VP8 was present in a reduced, but still significant, amount [22]. There was also a 100-fold reduction in the titer of BoHV-1YmVP8, which might be due to a substantial reduction in VP8 in the mature virus.

The fact that YmVP8 remained nuclear, and yet was present in the mature virion, led us to hypothesize that early packaging of VP8 occurs during the primary tegumentation process. We determined that VP8 is a part of the primary tegument of the primary enveloped virus, along with VP22—a small tegument protein translated by the *U_L_49* gene [23]. During infection, BoHV-1 and HHV-1 VP22 are known to localise into the nucleus with the help of an NLS present at the C-terminus [24,25]; however, other subcellular localisations are slightly different in HHV-1 and BoHV-1 [26]. We demonstrated that VP8 and VP22 interact and co-localize in the perinuclear region in cells infected with wild-type BoHV-1 or BoHV-1-YmVP8. VP22 was shown to be critical for the packaging of VP8 at the early tegumentation stage, as in VP22-deleted virus (BoHV-1ΔUL49)-infected cells the primary enveloped virions were found to be devoid of VP8.

## 2. Materials and Methods

### 2.1. Cell Lines and Viruses

COS-7 and Madin–Darby bovine kidney (MDBK) cells were grown in Dulbecco’s modified Eagle’s medium (DMEM; Sigma-Aldrich Canada Ltd., Oakville, ON, Canada) and Eagle’s minimum essential medium (MEM; Sigma-Aldrich Canada Ltd.), respectively, supplemented with 10 mM N-2-hydroxyethylpiperazine-N-2-ethane sulfonic acid (HEPES, Gibco, Life Technologies, Burlington, ON, Canada), 1% nonessential amino acids (Gibco, Life Technologies), 50 µg/mL gentamycin (Gibco, Life Technologies), and 10% fetal bovine serum (FBS; Gibco, Life Technologies), with 5% CO_2_ in a 37 °C incubator.

Wild-type BoHV-1, BoHV-1YmVP8, in which all of the phosphorylation sites were mutated [22], and BoHV-1ΔUL49—a VP22-deleted mutant [23]—were propagated and titred on MDBK cells in 24-well plates, followed by overlay with 0.8% UltraPure low-melting-point agarose (Invitrogen/Thermo Fisher Scientific, Waltham, MA, USA) in MEM, and stocks were maintained at −80 °C.

For viral infections, 85–90% confluent monolayers of MDBK cells were infected with BoHV-1, BoHV-1-YmVP8, or BoHV-1ΔUL49 at different multiplicities of infection (MOIs) in MEM. Virus was replaced after 1.5 h with MEM containing 2% FBS. Cells were collected at different timepoints post-infection.

### 2.2. Plasmids and Antibodies

The plasmid pFLAG-VP8 was previously described in [16]. pHA-VP22 was constructed by amplifying the *U_L_49* gene from the BoHV-1 genome using 3′ATTGAATTCATGGCCCGGTTCCACAGG 5′ and 3′ATTTCTAGAATTTTCATACTAGCA CAGCA 5′ primers, followed by ligation with an XbaI- and EcoRI-digested pcDNA3.1HA (Addgene, Watertown, MA, USA). Plasmid DNA was sequenced at the NRC Plant Biotechnology Institute (Saskatoon, SK, Canada).

Antibodies including mouse monoclonal VP8-specific antibody (clone 1G4 2G2), rabbit polyclonal VP22-specific antibody, and rabbit polyclonal VP5-specific antibody were described previously in [15,23]. FLAG tag-specific mouse monoclonal antibodies (Sigma-Aldrich Canada Ltd.) and HA tag-specific rabbit polyclonal antibodies (Cell Signaling Technology, Danvers, MA, USA) were used to detect the respective proteins. Mouse monoclonal antibodies specific to lamin-associated proteins A and C (LAP2) were purchased from Abcam Inc. (Toronto, ON, Canada). Mouse monoclonal antibodies specific to nucleolin, and rabbit polyclonal antibodies specific for tubulin, were purchased from Sigma-Aldrich Canada Ltd. For Western blotting, IRDye 680RD goat anti-rabbit IgG, IRDye 680RD goat anti-mouse IgG, IRDye 800RD goat anti-rabbit IgG, and IRDye 800RD goat anti-mouse IgG (Li-Cor Biosciences, Lincoln, NE, USA) were used. Alexa 488-conjugated goat anti-mouse IgG, Alexa 488-conjugated goat anti-rabbit IgG, Alexa 633-conjugated goat anti-mouse IgG, and Alexa 633-conjugated goat anti-rabbit IgG (Invitrogen/Thermo Fisher Scientific) were used for immunofluorescent staining.

### 2.3. Isolation of Nuclear Membrane

The procedure for the isolation of the nuclear membrane was derived from a procedure applied to HHV-1-infected COS-7 cells [27]. MDBK cells were infected at an MOI of 10 with wild-type BoHV-1 or BoHV-1ΔUL49 and incubated at 37 °C for 8 h. Cells were collected in sucrose buffer A (10 mM Tris-HCl pH 7.4, 0.32 M sucrose, 3 mM MgCl_2_) and centrifuged at 1700× *g* for 10 min, followed by resuspension of the pellets in fresh sucrose buffer A with 60 µL/mL of protease inhibitor cocktail (Sigma-Aldrich Canada Ltd.). Cells were manually disrupted using a 25G × 5/8”-gauge needle; the resulting suspension was termed the total cell extract (TCE). The TCE was centrifuged for 10 min at 700× *g*, and the nuclei in the pellet were resuspended in sucrose buffer B (10 mM Tris-HCl pH 7.4, 2.4 M sucrose, 3 mM MgCl_2_) and centrifuged at 50,000× *g* for 1 h at 4 °C. The resulting pellet contained the purified nuclei, which were washed and resuspended in 1 mM DNase and digestion buffer (10 mM Tris-HCl pH 8.5, mM β-mercaptoethanol, 0.3 M sucrose), and then incubated for 20 min at room temperature. The washed nuclei were centrifuged at 40,000× *g* for 15 min, and the digestion was repeated for 1 h, followed by centrifugation at 40,000× *g* for 15 min. The resulting pellet contained the nuclear membrane and was called the nuclear membrane preparation (NMP) and resuspended in sucrose buffer C (10 mM Tris-HCl pH 7.4, 0.25 M sucrose, 3 mM MgCl_2_).

### 2.4. Isolation of Primary Enveloped Virions

The NMP was sonicated (5 pulses of 10 s on and 20 s off) in order to release the primary enveloped virions from the perinuclear space. The suspension was incubated with 50 µg/mL of proteinase K (Sigma-Aldrich, Canada Ltd.) for 1 h on ice, and quenched with 1 mM phenylmethylsulfonyl fluoride (PMSF) (Sigma-Aldrich, Canada Ltd.). The suspension was then overlaid on a 30% sucrose cushion (Sigma-Aldrich, Canada Ltd.) (in 10 mM Tris-HCl, pH 7.4) and centrifuged at 40,000× *g* for 1 h at 4 °C. The pellet containing the primary enveloped virions was resuspended in sucrose buffer C.

### 2.5. Purification of Mature Virus

MDBK cells were infected with wild-type BoHV-1 or BoHV-1ΔUL49 at an MOI of 10, and culture medium was collected 18 h post-infection. The medium was clarified at 3000× *g* for 20 min to remove any cell debris. The supernatant was collected and overlaid over 30% sucrose (in TNE (10 mM Tris-HCl pH 7.4, 1.5 mM NaCl, 1 mM EDTA)), and was centrifuged for 2 h at 25,000× *g* and 4 °C. The resulting viral pellet was solubilised at 4 °C in TNE overnight. The virus was layered over a 20–60% sodium potassium tartrate gradient and centrifuged for 90 min at 28,000× *g* and 4 °C; this step was performed twice. The virus band was collected, diluted in TNE, and centrifuged at 25,000× *g* and 4 °C for 1 h. The resulting pellet was resuspended in TNE and stored at −80 °C until further use.

### 2.6. Gel Electrophoresis

Twenty microliters of both the primary enveloped virus particles and the purified mature virions were run on an 8% SDS-polyacrylamide gel after being boiled in SDS loading dye at 95 °C for 3 min. The gel was stained with ProtoBlue Safe (National Diagnostic, Thermo Fisher Scientific) and de-stained in distilled water.

### 2.7. Mass Spectrometry Sample Preparation and Analysis

The viral samples purified from culture medium (mature) and the perinuclear space (primary enveloped) were precipitated using −20 °C cold acetone. Two sets of samples from two different infections were sent to the Mass Spectrometry Facility at the University of Missouri (Columbia, MI, USA). Acetone-precipitated samples from the mature and primary enveloped virus particles were washed and resuspended with 6 M urea, 2 M thiourea, and 100 mM ammonium bicarbonate. In-solution digestion was carried out overnight and then stopped after 16 h. The samples were then zip-tipped, lyophilised, resuspended, and loaded into the LTK Orbitrap XL (Thermo Fisher Scientific). Subsequently, proteins were identified using computer software against the NCBI Herpesvirus Database.

### 2.8. Transfections

Monolayers of COS-7 cells at 80–90% confluency were transfected with a total of 3 µg of plasmid (pFLAG-VP8, pVP22-HA, or pFLAG-VP8 + pVP22-HA) using Lipofectamine LTX transfection reagent (Invitrogen/Thermo Fisher Scientific), and cells were collected 24–48 h post-transfection.

### 2.9. Preparation of Cell Lysates

Infected MDBK cells or transfected COS-7 cells were collected using ice-cold phosphate-buffered saline at pH 7.4 (PBS) (Gibco, Life technologies) after various time intervals, and then centrifuged at 8000× *g* for 10 min. Cell pellets were subjected to lysis in RIPA buffer (50 mM Tris-HCl, 150 mM NaCl, 1mM EDTA, 1% Triton-X100, pH 7.4) and protease inhibitor cocktail (Sigma-Aldrich Canada Ltd.) at a 10:1 *v*/*v* ratio for 40 min on ice. Cell lysates were clarified by centrifugation at 12,000× *g* for 10 min at 4 °C. The supernatants were collected and stored at −20 °C for further use.

### 2.10. Immunoprecipitation and Western Blotting

Infected cell lysates (prepared as described above) were incubated with VP8-specific or VP22-specific antibodies overnight at 4 °C, followed by Protein G Sepharose Fast Flow beads (GE HealthCare, Niskayuna, NY, USA) for 3 h at 4 °C. Transfected cell lysates were incubated with Anti-FLAG M2 Affinity Gel (Sigma-Aldrich Canada Ltd.) or anti-HA agarose (Pierce/Thermo Fisher Scientific) and incubated at 4 °C overnight. Lysates were washed 3–4 times with wash buffer (0.05 M Tris-HCl, 0.15 M NaCl, pH 8) and eluted with SDS loading dye by boiling at 95 °C for 2 min. SDS loading dye was also added to a fraction of the whole cell lysates, which were then boiled at 95 °C for 2 min. These samples were subjected to SDS-polyacrylamide gel electrophoresis on 8% gels, and transferred to 0.45 µm nylon membranes (Bio-Rad, Hercules, CA, USA). This was followed by blocking the membrane with 3% blotto (3 g milk powder in TBST (20 mM Tris-HCl, 150 mM NaCl, 0.1% *w*/*v* Tween 20, pH 7.4) for 1 h, and incubation with the corresponding antibodies overnight. The next day, the membranes were washed with TBST and incubated with IRDye 680RD goat anti-mouse IgG or IRDye 800RD goat anti-rabbit IgG (LI-COR Bioscience, Lincoln) for 2 h. An Odyssey Infrared Imaging System and Odyssey 3.0.16 application software (LI-COR Biosciences) were used for detection and analysis.

### 2.11. Confocal Microscopy

Cells were seeded in 2-well Permanox Lab-Tek chamber slides (Nunc, Thermo Fisher), and were infected at 80–90% confluency with BoHV-1, BoHV-1YmVP8, or BoHV-1ΔUL49 at various MOIs. Cells were fixed at different times post-infection with 4% paraformaldehyde (Sigma-Aldrich Canada Ltd.) and washed with phosphate-buffered saline (10 mM Na_2_HPO_4_, 1.8 mM KH_2_PO_4_, 137 mM NaCl, 2.7 mM KCl, pH 7.4 (PBS)). After washing, the cells were blocked overnight with 1% goat serum. The following day, the cells were treated with 0.1% Triton X-100 to permeabilize them. Subsequently, they were incubated with the corresponding primary antibodies prepared in 1% goat serum for 2–3 h, followed by Alexa 488-conjugated goat anti-rabbit or goat anti-mouse IgG, Alexa 633-conjugated goat anti-rabbit or goat anti-mouse IgG, or both Alexa 488- and Alexa 633-conjugated IgG (Invitrogen, Thermo Fisher), for 1–2 h at room temperature. Prolong Gold with DAPI (Invitrogen, Thermo Fisher) was used as a mounting medium and for staining of the nuclei of the cells after the antibody incubations. These cells were analysed with a Leica SP8 confocal microscope (Leica Microsystems, Wetzlar, Germany), and Leica Application Suite X software was used to enlarge cells to indicate localisations in detail. Perinuclear localization of VP8 and/or VP22 was enumerated in 10 fields with 30–100 cells each.

## 3. Results

### 3.1. VP8 and VP22 Are Packaged at the Early Tegumentation Stage

**Figure 1:**
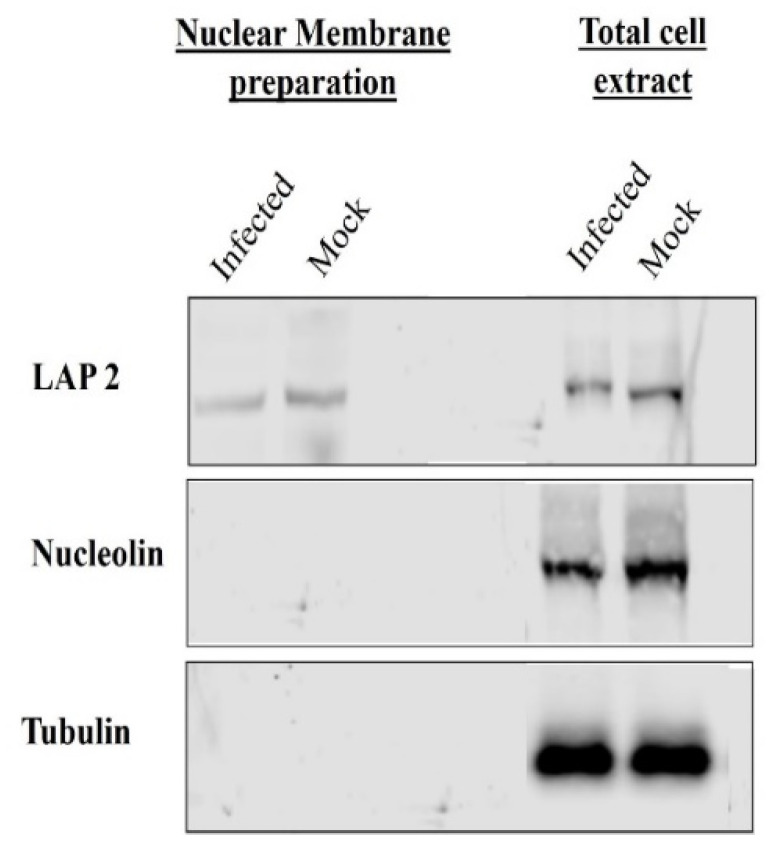
Nuclear membrane preparations from BoHV-1-infected and mock-infected MDBK cells were separated by SDS-PAGE on 8% reducing gels and subjected to Western blotting to check for the absence of nucleolin and tubulin, and the presence of LAP2, in the nuclear membrane. The cellular markers were detected by mouse monoclonal anti-LAP2, mouse monoclonal anti-nucleolin, and rabbit polyclonal anti-tubulin IgG, followed by IRDye 680RD goat anti-mouse IgG and IRDye 680RD goat anti-rabbit IgG, respectively.

**Figure 2:**
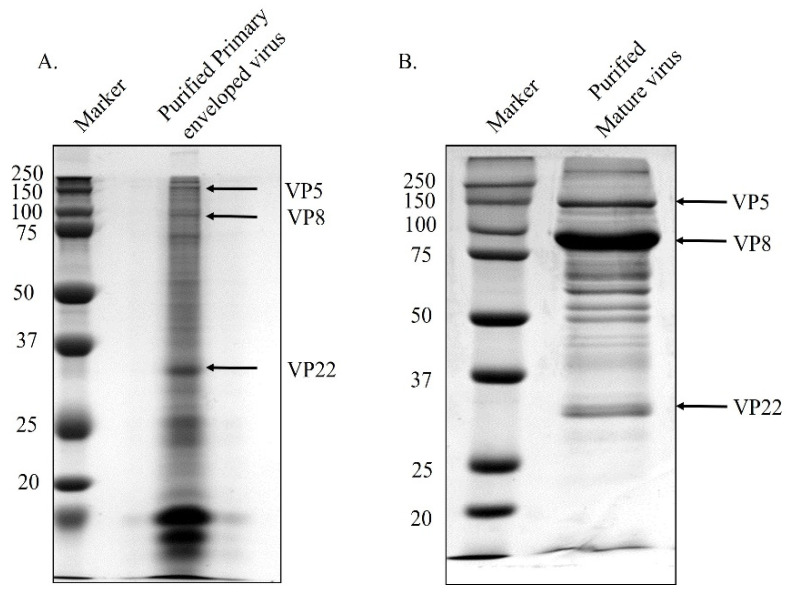
Purified primary enveloped virus (**A**) and mature virus (**B**). MDBK cells were infected with BoHV-1, and virus was purified from the perinuclear membrane at 8 h post-infection, and from the culture medium at 18 h post-infection. The proteins were separated by SDS-PAGE on 8% reducing gels and stained with ProtoBlue Safe. The positions of the capsid protein VP5 and tegument proteins VP8 and VP22 are indicated with arrows. Molecular weight markers (MWs) ×10^−3^ are shown in the left margin.

When not phosphorylated, VP8 remains nuclear during viral infection, but is present in a significant amount in the mature BHV-1YmVP8 virus particles. In order to examine the possibility of VP8 being packaged at the early tegumentation stage, primary enveloped virions were isolated from the perinuclear region and tested for the presence of VP8 by mass spectrometry and Western blotting. Prior to the extraction of the primary enveloped virus from the perinuclear membranes, the purity of the NMP was tested. Both TCE and NMP were analysed for the presence of tubulin, a cytoskeletal protein; nucleolin, a nuclear protein; and lamin-associated protein 2 (LAP2), a protein specific to the nuclear membrane. The NMP was found to be devoid of tubulin and nucleolin; only LAP2 was found in the NMP, confirming it to be free from other cellular components (Figure 1). To determine their purity, the mature and primary enveloped viruses were analysed via SDS-PAGE. Figure 2 shows the stained polyacrylamide gel, which indicates distinct bands of VP8 (91 kD) and VP22 (29 kD) in both mature and primary enveloped virus samples. The detection of VP5 (the major capsid protein; 105 kD) confirms the presence of virus particles.

**Figure 3:**
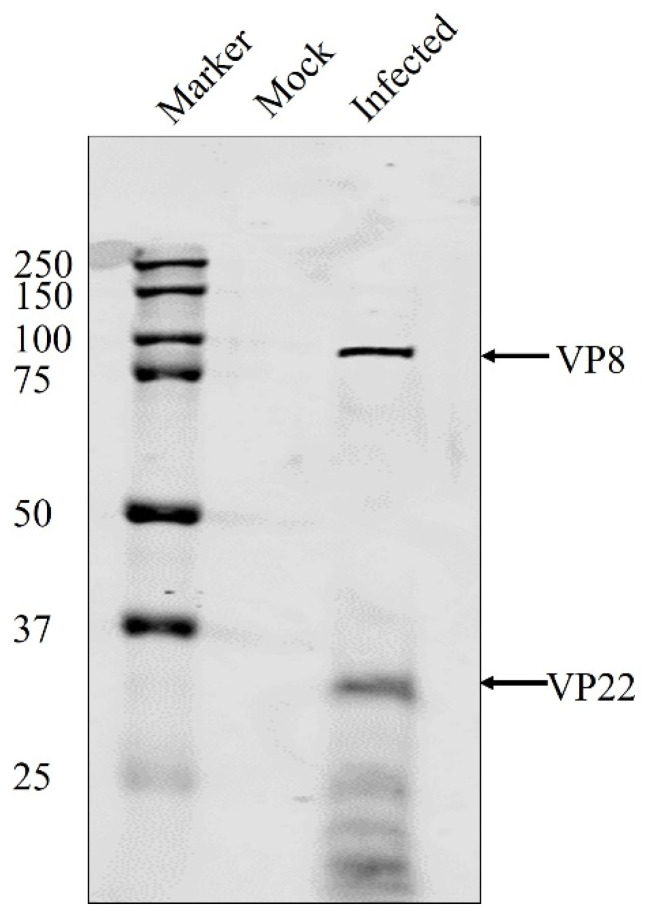
Identification of VP8 and VP22 in primary enveloped virus purified from the NMP of MDBK cells infected with wild-type BoHV-1 at an MOI of 10 and harvested 7 h post-infection. The proteins were separated by SDS-PAGE on 8% reducing gels, and VP8 and VP22 were identified by Western blotting with mouse VP8-specific monoclonal antibody and rabbit VP22-specific polyclonal antibody, followed by IRDye 680RD goat anti-mouse IgG and IRDye 800RD goat anti-rabbit IgG, respectively. The positions of the tegument proteins VP8 and VP22 are indicated with arrows. Molecular weight markers (MWs) ×10^−3^ are shown in the left margin.

Viruses purified from the culture medium and the perinuclear membrane were submitted to mass spectrometry analysis. Table 1 shows a comprehensive list of the mass spectrometry results. As hypothesised, VP8 was found to be a part of the primary enveloped virus, suggesting its packaging at the early stage of tegument formation. Interestingly, another tegument protein—VP22—was also found to be a part of the early tegument. In HHV-1, VP22 is known to play a critical role in viral protein packaging, while in BoHV-1 it is known to aid in the packaging of gN, so VP22 might play a role in VP8 packaging. The presence of both VP8 and VP22 in primary enveloped virus particles was confirmed by Western blotting (Figure 3).

### 3.2. VP8 Interacts with VP22 in the Perinuclear Region and in Mature Virus in BoHV-1-Infected Cells and in BoHV1-YmVP8-Infected Cells

**Figure 4:**
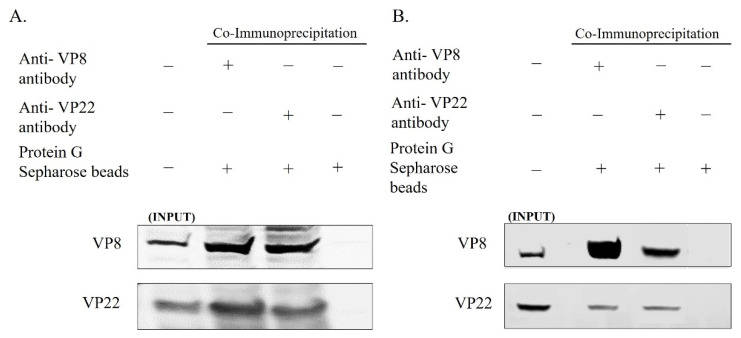
VP8 and VP22 interact during BoHV-1 infection. MDBK cells were infected with wild-type BoHV-1 at an MOI of 3 and harvested 8 h post-infection (primary enveloped virus; (**A**)) or 18 h post infection (mature virus; (**B**)). Lysates were generated and incubated with mouse VP8-specific monoclonal antibodies or rabbit VP22-specific polyclonal antibodies, followed by Protein Sepharose G beads. VP8 and VP22 were detected by Western blotting with monoclonal anti-VP8 and rabbit anti-VP22 antibodies, followed by IRDye 680RD goat anti-mouse IgG and IRDye 800RD goat anti-rabbit IgG, respectively. Immunoprecipitation of VP8 with VP22—and vice versa—is shown for both primary enveloped (**A**) and mature (**B**) virus.

In view of the coexistence of VP8 and VP22 in the primary enveloped virus, and the fact that the VP22 levels were reduced in the mature virus when the cells were infected with BoHV-1ΔUL47, we studied the interaction between VP8 and VP22 in the mature and primary enveloped virus particles. The viruses collected from the culture medium and the NMP of MDBK cells infected with wild-type BoHV-1 were lysed and incubated overnight with antibodies specific to VP8 or VP22, followed by incubation with Protein G Sepharose. After the bound proteins were eluted and subjected to Western blotting, it was found that VP22 interacted with VP8—and vice versa—in the primary enveloped virions obtained from the NMP (Figure 4A). Similarly, in the mature virus, VP22 interacted with VP8, and vice versa (Figure 4B).

**Figure 5:**
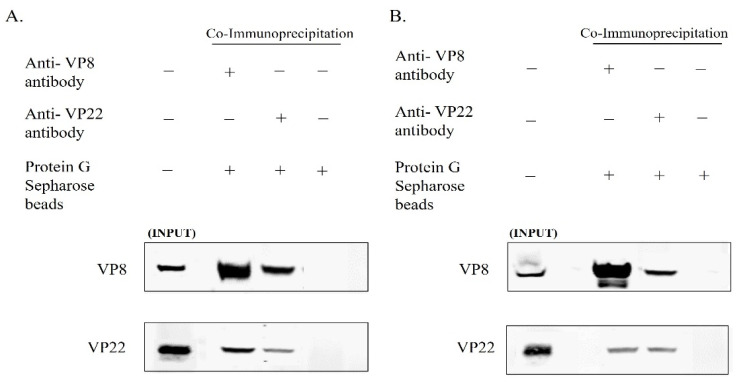
VP8 and VP22 interact during BoHV-1YmVP8 infection. MDBK cells were infected with BoHV-1YmVP8 at an MOI of 0.5 and harvested 18 h post-infection (primary enveloped virus; (**A**)) or 30 h post-infection (mature virus; (**B**)). Lysates were generated and incubated with mouse VP8-specific monoclonal antibodies or rabbit VP22-specific polyclonal antibodies, followed by Protein Sepharose G beads. VP8 and VP22 were detected by Western blotting with monoclonal anti-VP8 and rabbit anti-VP22 antibodies, followed by IRDye 680RD goat anti-mouse IgG and IRDye 800RD goat anti-rabbit IgG, respectively. Immunoprecipitation of VP8 with VP22—and vice versa—is shown for primary enveloped (**A**) and mature (**B**) virus.

In order to determine whether VP8 and VP22 interact in cells infected with BoHV-1-YmVP8, in which VP8 is not phosphorylated and remains nuclear, MDBK cells were infected with BoHV-1-YmVP8 at an MOI of 0.5 and collected at 18 h post-infection to purify the perinuclear membrane, and at 30 h post-infection to isolate mature virus. The cell lysates were incubated overnight with VP8-specific or VP22-specific antibodies, followed by binding to Protein G Sepharose beads and elution with SDS loading dye. Western blot analysis showed coprecipitation of VP22 with VP8—and vice versa—in the NMP (Figure 5A). Similarly, in the mature virus, VP22 interacted with VP8, and vice versa (Figure 5B).

These data suggest that VP8 and VP22 interact with one another in the NMP, and possibly at a later stage during final envelopment at the Golgi. The interaction with VP22 does not depend on phosphorylation of VP8, as it occurred with both the wild-type VP8 and the non-phosphorylated form of VP8.

### 3.3. VP8 Is Absent in the Primary Enveloped Virus in the Absence of VP22

**Figure 6:**
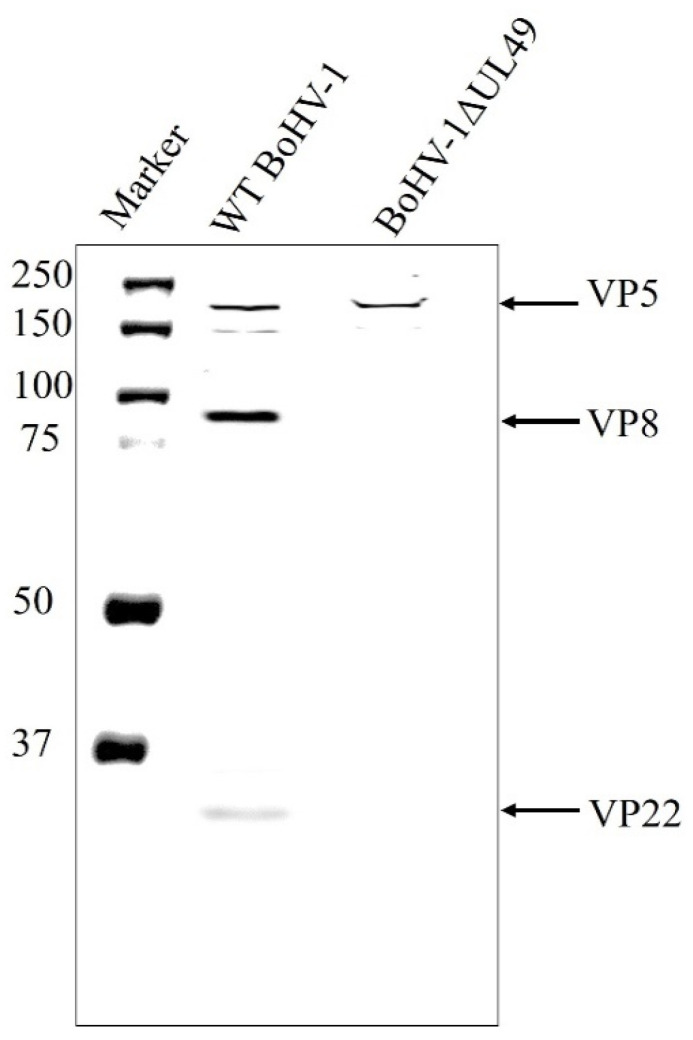
VP8 is absent in primary enveloped virus in BoHV-1ΔUL49-infected cells. Primary enveloped virus was purified from the NMP of MDBK cells infected with BoHV-1ΔUL49 at an MOI of 3, and harvested 18 h post-infection. The proteins in the primary enveloped virus were separated by SDS-PAGE on 8% gels, and VP8 and VP22 were identified by Western blotting with mouse VP8-specific monoclonal antibody and rabbit VP22-specific polyclonal antibody, followed by IRDye 680RD goat anti-mouse IgG and IRDye 800RD goat anti-rabbit IgG, respectively. The positions of the tegument proteins VP8 and VP22 are indicated with arrows. Molecular weight markers (MWs) × 10^−3^ are shown in the left margin.

Since an interaction was found between VP8 and VP22, the dependence of VP8 on VP22 for its packaging into the primary tegument was examined. MDBK cells were infected with wild-type BoHV-1 or BoHV-1ΔUL49, and the virions extracted from the NMP were tested for the presence of VP8 by Western blotting. VP8 was not detected in the NMP of the BoHV-1ΔUL49-infected cells, suggesting that VP22 is required for the packaging of VP8 during primary envelopment. VP5 (a major capsid protein) was used as a control to confirm the presence of virus particles (Figure 6).

### 3.4. VP8 and VP22 Interact with one Another Outside the Context of Infection

**Figure 7:**
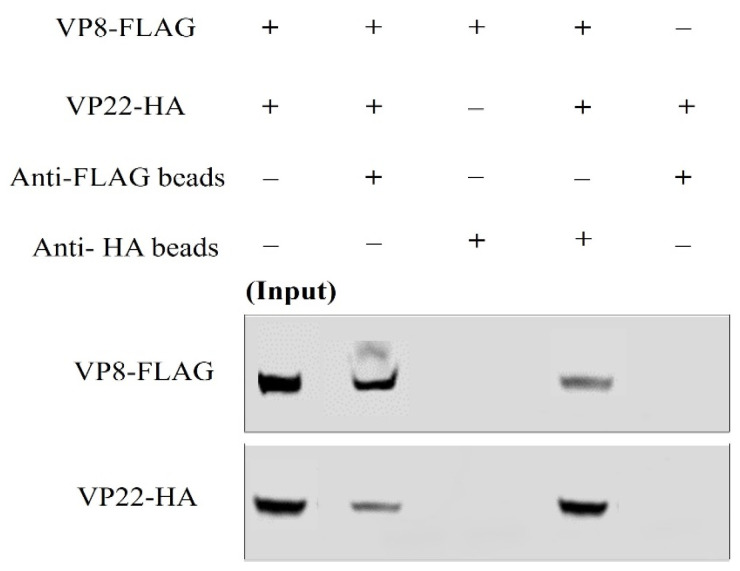
VP8 and VP22 interact outside the context of infection in transfected cells. COS-7 cells were transfected with VP8-FLAG and/or VP22-HA plasmids. The cells were lysed at 24 h post-transfection, and incubated with Anti-FLAG M2 Affinity Gel or anti-HA agarose. VP8 and VP22 were detected by Western blotting with monoclonal mouse anti-FLAG and polyclonal rabbit anti-HA antibodies, followed by IRDye 680RD goat anti-mouse IgG and IRDye 800RD goat anti-rabbit IgG, respectively. VP22 was precipitated with VP8 and Anti-FLAG M2 Affinity Gel, while VP8 was precipitated with VP22 and anti-HA agarose.

In order to determine whether the interaction between VP8 and VP22 is independent of other viral factors, COS-7 cells were co-transfected with both VP8-FLAG and VP22-HA plasmids (and individually with VP8-FLAG and VP22-HA as controls). At 48 h after transfection the cells were collected and lysed. The lysates were incubated with anti-FLAG beads or anti-HA beads, and the proteins were eluted with SDS loading dye. When these samples were subjected to Western blotting, it was found that VP8-FLAG was precipitated along with VP22-HA when the lysate was incubated with anti-HA beads, and that VP22-HA was precipitated along with VP8-FLAG when the lysate was incubated with anti-FLAG beads. The specificity of the VP8-FLAG and VP22-HA interaction was proven by the absence of VP8-FLAG when the lysate from VP8-FLAG-transfected cells was incubated with anti-HA agarose, and similarly, the absence of VP22-HA when the lysate from VP22-HA-transfected cells was incubated with Anti-FLAG M2 Affinity Gel (Figure 7).

### 3.5. VP8 and VP22 Localise in the Perinuclear Region in Cells Infected with Wild-Type BoHV-1 or BoHV-1YmVP8

**Figure 8:**
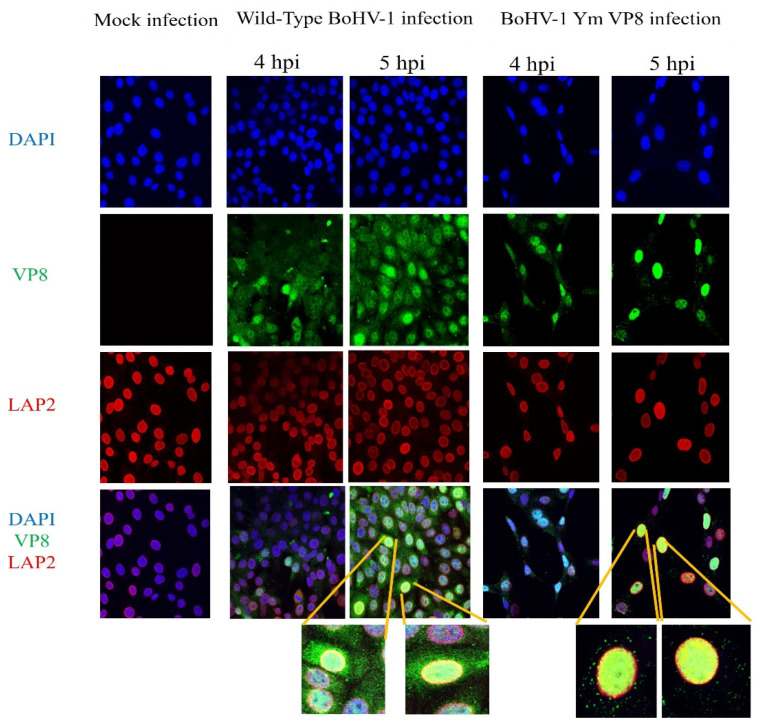
Co-localisation of VP8 and LAP2. MDBK cells were infected with wild-type BoHV-1 or BoHV-1YmVP8 at an MOI of 5, and fixed and permeabilised at 4 or 5 h post-infection. VP8 was identified with rabbit VP8-specific antibody and Alexa-488-conjugated goat anti-rabbit IgG. LAP2 was detected with monoclonal LAP2-specific antibody and Alexa-633 conjugated anti-mouse IgG. The yellow ring around the cells shows VP8 co-localised with LAP2 in the perinuclear region. Select cells were observed at 10× magnification to show more details.

To confirm the presence of VP8 and VP22 in the perinuclear region at the early stages of infection, MDBK cells were infected with wild-type BoHV-1 or BoHV-1-YmVP8 at an MOI of 5 and fixed at 4 or 5 h post-infection. Antibodies specific to LAP2, followed by Alexa 633-conjugated goat anti-mouse IgG, were used to identify the nuclear membranes and antibodies specific to VP8 and VP22, followed by the use of Alexa 488-conjugated goat anti-rabbit IgG to identify the respective proteins. In both wild-type BoHV-1- and BoHV-1YmVP8-infected cells, the presence of VP8 in the perinuclear nuclear region was confirmed by the yellow fluorescence. No signal was observed in mock-infected cells. Figure 8 shows co-localisation of VP8 with LAP2 in the perinuclear region of MDBK cells infected with wild-type BoHV-1 or BoHV-1YmVP8, at 4 and 5 h post-infection. At 4 h post infection, VP8 was present at low levels, and was mostly nuclear in both wild-type BoHV-1- and BoHV-1YmVP8-infected cells. After infection with wild-type BoHV-1, 2.6% of the cells showed the presence of VP8 in the perinuclear region. In BoHV-1YmVP8-infected cells, less co-localisation of VP8 and LAP2 (<2%) was observed when compared to the wild-type BoHV-1-infected cells. At 5 h post-infection, VP8 expression increased; in wild-type BoHV-1-infected cells, a low proportion of VP8 was cytoplasmic, while 37.5% of the observed cells showed the presence of VP8 in the perinuclear region. Meanwhile, in BoHV-1YmVP8-infected cells, VP8 remained nuclear due to the absence of phosphorylation; in 36.74% of the cells, a distinct co-localisation of VP8 with LAP2 was observed in the perinuclear region. These localisation patterns continued through the later stages of infection (data not shown). This supports the hypothesis of a certain amount of VP8 being packaged during the early envelopment process in both BoHV-1- and BoHV-1YmVP8-infected cells.

**Figure 9:**
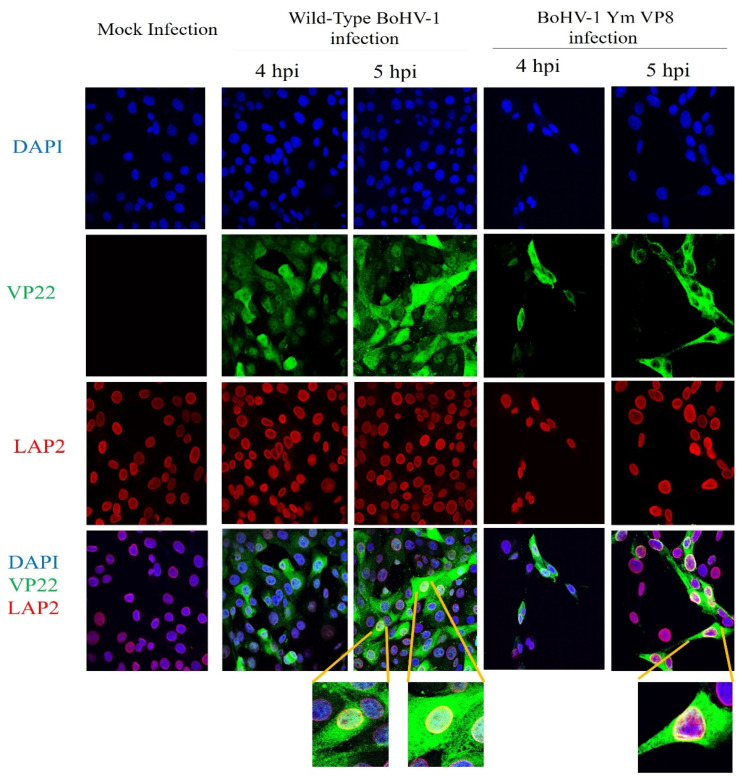
Localisation of VP22 in the perinuclear region. MDBK cells were infected with wild-type BoHV-1 or BoHV-1YmVP8 at MOI 5, and fixed and permeabilized 4 or 5 h post-infection. VP22 was identified with rabbit VP22-specific antibody and Alexa-488-conjugated goat anti-rabbit IgG. LAP2 was detected with monoclonal LAP2-specific antibody and Alexa-633 conjugated anti-mouse IgG. The yellow ring around the cells shows VP22 co-localised with LAP2 in the perinuclear region. Select cells were observed at 10× magnification to show more details.

Figure 9 shows similar results for the presence of VP22 in the perinuclear region. VP22 was mostly cytoplasmic at 4 h post-infection in both BoHV-1- and BoHV-1YmVP8-infected cells. However, at 4 h post-infection, 9% of the BoHV-1-infected cells were found to have VP22 present in the perinuclear region. In BoHV-1YmVP8-infected cells, 15% of the cells showed VP22 localisation in the perinuclear region. Localisation of VP22 in the perinuclear region was evident at 5 h post-infection in both BoHV-1- and BoHV-1YmVP8-infected cells. After wild-type BoHV-1 infection, 31.6% of the cells showed VP22 in the perinuclear region, whereas in BoHV-1YmVP8 infected cells 38.5% showed a clear presence of VP22 in the perinuclear region. No signal was observed in mock-infected cells. VP22 is predominantly cytoplasmic during the early stages of infection [28]; however, due to its small size, VP22 (29 kDa) can passively diffuse into the nucleus via the nuclear pores [29,30], which is probably responsible for some nuclear VP22.

### 3.6. VP22 Co-Localises with VP8

**Figure 10:**
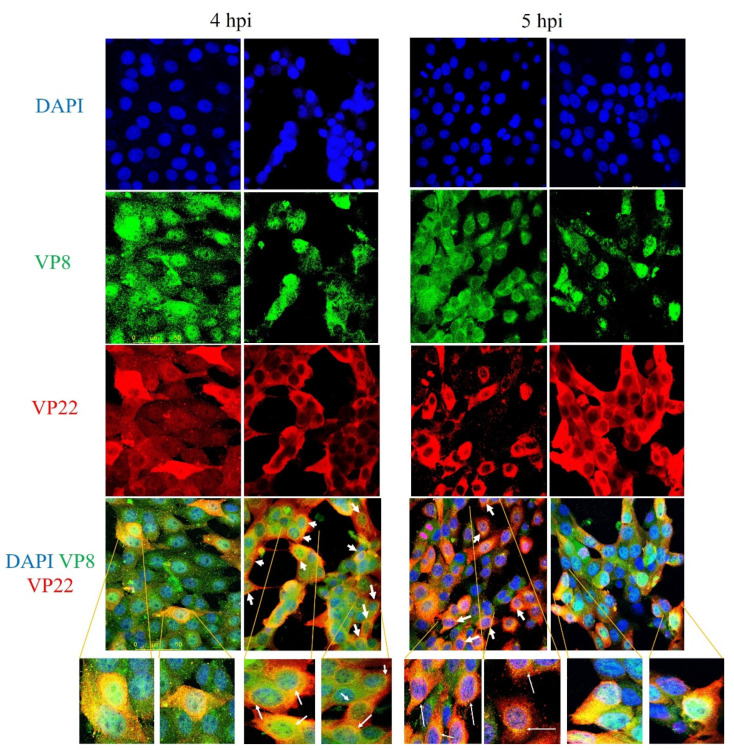
Co-localisation of VP22 and VP8. MDBK cells were infected with wild-type BoHV-1, and fixed and permeabilized 4 or 5 h post-infection. VP8 was identified with monoclonal VP8-specific antibody and Alexa 488-conjugated goat anti-mouse IgG. VP22 was detected with rabbit VP22-specific antibody and Alexa 633-conjugated anti-rabbit IgG. The yellow ring (as indicated by the arrows) shows co-localisation of VP8 and VP22 proteins in the perinuclear region. Select cells were observed at 10× magnification to show more details.

After proving the interaction of VP8 and VP22, and the presence of VP8 and VP22 in the perinuclear region, co-localisation of VP8 and VP22 was investigated in wild-type BoHV-1-infected cells at an MOI of 5. Figure 10 shows co-localisation of VP8 and VP22 proteins at 4 h and 5 h post-infection. At 4 h post-infection, co-localisation of VP8 and VP22 was observed in 15% of the cells—mostly around the perinuclear region, with some in the cytoplasm. At 5 h post-infection, more co-localisation of VP8 and VP22 was observed in the cytoplasm than in the perinuclear region, but 38.5% of cells showed co-localisation of VP8 and VP22 in the perinuclear region.

### 3.7. VP8 Is not Localised to the Perinuclear Region in BoHV-1ΔUL49-Infected Cells

**Figure 11:**
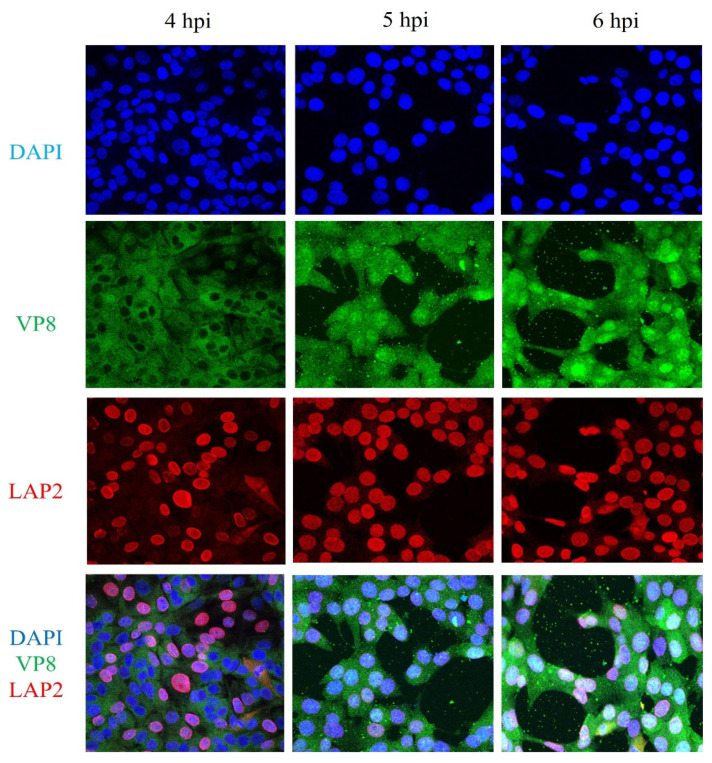
VP8 does not localise in the perinuclear region in BoHV-1ΔUL49- infected cells. MDBK cells were infected with BoHV-1ΔUL49 at an MOI of 5, and fixed and permeabilized 4, 5, or 6 h post infection. VP8 was identified with rabbit VP8-specific antibody and Alexa 488-conjugated goat anti-rabbit IgG. LAP2 was detected with monoclonal LAP2-specific antibody and Alexa 633-conjugated anti-mouse IgG. No yellow and, hence, no presence of VP8 in the perinuclear region, was detected.

To further confirm the absence of VP8 in the perinuclear region of BoHV-1ΔUL49-infected cells, as shown in Figure 6, MDBK cells were infected with BoHV-1ΔUL49 at an MOI of 5 and fixed 4 or 5 h post-infection. LAP2-specific antibodies, followed by Alexa 633-conjugated goat anti-mouse IgG, were used to identify the nuclear membranes and antibodies specific to VP8, followed by the use of Alexa 488-conjugated goat anti-rabbit IgG to identify VP8. In BoHV-1ΔUL49-infected cells, no nuclear VP8 was detected at 4 h post-infection, which may be due to the slower rate of BoHV-1ΔUL49 infection, whereas at 5 and 6 h post-infection VP8 was partially nuclear and partially cytoplasmic—similar to wild-type BoHV-1-infected cells. No VP8 was localised in the perinuclear region at 4, 5, or 6 h post-infection (Figure 11). From these results, it can be inferred that VP22 is required for the perinuclear localisation and, hence, the packaging of VP8 during the early tegumentation process.

## 4. Discussion

In this study, a novel, VP22-dependent packaging mechanism of VP8 during primary envelopment was identified. During the maturation process of alpha herpesviruses, tegument proteins are added at different stages—namely, during primary envelopment, i.e., when the virus moves from the nucleus into the perinuclear region during its egress [12]; in the cytoplasm, after it exits the nucleus and travels towards the Golgi; and at the Golgi, where the final maturation takes place [7,11]. VP8 contains phosphorylation sites, and is phosphorylated by host kinase CK2 in the cytoplasm, and by viral kinase US3 in the nucleus [31]. Previous studies showed that the phosphorylation of VP8 by US3 is necessary for its translocation into the cytoplasm, as well as for various other functions [22]. Tegument proteins such as VP8 may be completely packaged during the primary envelopment process, completely packaged at the Golgi, or partially packaged during primary envelopment and partially in the Golgi. A similar pattern of packaging during the early tegumentation process has been shown in previous studies for HHV-1 VP22 [32]. When MDBK cells were infected with BoHV-1YmVP8 and the mature virus was tested for the amount of VP8, a significant amount of VP8 was found to be present in the BoHV-1YmVP8 virions, despite the fact that VP8 remained nuclear [22]. This suggested that VP8 might be packaged during early tegumentation; however, due to the fact that the amount of VP8 in the mature BoHV-1YmVP8 virions was significantly reduced, it can be inferred that the early packaging of VP8 at the nucleus is only partial, and that VP8 is further packaged in the Golgi.

In this study, mass spectrometry analysis performed on biological duplicate samples of primary enveloped virions extracted from the NMP and the mature virus extracted from the culture medium identified the proteins specific to these fractions. Based on the purity of the fractions, it can be inferred that the NMP was devoid of any nuclear or cytoplasmic contaminants, and that the identified proteins were solely from the primary enveloped virus present in the perinuclear region. The comprehensive list of identified proteins is similar to the list of proteins present in HHV-1 for both primary enveloped and mature viruses [27,32,33]. In the BoHV-1 mass spectrometry analysis, 11 glycoproteins—gB, gC, gD, gE, gH, gL, gG, gM, gN, gK, and gI—were found to be a part of the mature virus, as glycoproteins are acquired at the Golgi along with the lipid envelope; this is consistent with previous studies on the protein composition of BoHV-1 [34]. The absence of glycoproteins from the primary enveloped virus further confirms the purity of the virus extracted from the perinuclear region. Major and minor capsid proteins were found to be a part of both the primary enveloped and the mature virus, which demonstrates the presence of the viral capsid in these regions. Most of the tegument proteins were found to be a part of only the mature virus—i.e., they are packaged outside the nucleus in the cytoplasm or at the Golgi—except for pUL2, pUL36, and pUL37, which were only present in the primary enveloped virions, meaning that they are packaged during the early tegumentation process, and are lost as the capsids travel from the nucleus to the Golgi. In HHV-1, pUL36 and pUL37 are known to play critical roles in nuclear egress and, hence, are only needed at the early stages [12]. pUL26, pUL29, pUL39, pUL40, pUL47 (VP8), and pUL49 (VP22) were found to be part of the primary enveloped virus as well as the mature virus, which suggests that they are packaged during early tegument formation, and retained and/or lost and added during travel to the Golgi. Host proteins—namely, HSP70, annexin, tubulin, actin, PCNA, and histones, known to form an integral part of herpesviruses [33]—were also found in the BoHV-1 mature virus samples and, in the case of histones and PCNA, also in the primary enveloped virus particles. The absence of annexin from the primary enveloped BoHV-1 is in contrast with HHV-1, in which annexin 2 was found to be an integral part of the primary enveloped virus [27]. Overall, fewer host proteins were found in the BoHV-1 virus particles when compared to HHV-1, which may be related to the purity of the samples or the specific characteristics of these herpesviruses.

VP8 and VP22 were both found to be a part of primary enveloped viruses, i.e., a certain amount of both proteins is packaged during the early tegumentation process. In HHV-1 and BoHV-1, VP22 is a critical tegument protein, which plays a role in the packaging of several other tegument proteins as well as glycoproteins [35,36]; it is also responsible for the shuttling of viral proteins in the host cells [37,38,39]. The viral titer was also reduced 10-fold in BoHV-1ΔUL49-infected cells when compared to wild-type BoHV-1-infected cells [23]. A reduction in the amount of VP22 in BoHV-1ΔUL47-infected cells [4] suggests interdependence between VP8 and VP22. Partial packaging of critical proteins such as VP8 and VP22 into the early tegument might also be a survival strategy of the virus under conditions that oppose the transportation or translocation of these proteins to the Golgi. In this study, an interaction between VP8 and VP22 was confirmed in both the primary enveloped virions and the mature virus. This interaction was independent of the phosphorylation status of VP8 (as they were found to interact in BoHV-1-YmVP8-infected cells), and also of other viral factors (as they interact outside the context of infection). The presence of VP8 and VP22 was shown in the perinuclear region at early timepoints, which supports the probability of both proteins being packaged during early tegumentation, and assisting one another in the process.

Because VP22 aids in the packaging of proteins, and interacts with VP8, the requirement of VP22 for VP8 packaging at the early tegumentation stage was investigated. The fact that in BoHV-1ΔUL49-infected cells VP8 was absent from the perinuclear region (Figure 6 and Figure 11) confirms that VP22 is needed for the packaging of VP8 into the primary enveloped virus particles.

In summary, our results support the hypothesis that VP8 is partially packaged during the early tegumentation process along with VP22, and that VP22—an interaction partner of VP8—is required for the early packaging of VP8 into the early tegument. In the absence of phosphorylation, when VP8 remains mostly nuclear, the limited quantity of VP8 packaged during the early tegumentation process helps the virus to obtain a certain amount of this critical and multifunctional protein. However, in wild-type virus-infected cells, most of the VP8 is translocated into the cytoplasm and, hence, gets packaged at the Golgi along with the partial packaging during the early tegumentation. In addition to suggesting a novel mechanism for VP8 packaging during the early tegumentation process, this study also supports the correlation of the amount of VP8 in the virions with the replication efficiency in cell culture. Based on previous studies and the current study, it can be safely said that VP8 plays a critical role in viral replication, and that its reduction in mature virus causes the replication efficiency and virulence to be significantly reduced.

## 5. Conclusions

The presence of both VP8 and VP22 as a part of the early tegument indicates the early packaging of these proteins. This explains why a significant, though reduced, amount of VP8 is incorporated in the virus particles when it is not phosphorylated and remains nuclear. In cells infected with a VP22-deleted mutant, VP8 was not present in the early tegument, which suggests that VP8 depends on VP22 for its early packaging.

## Figures and Tables

**Table 1:** Comprehensive list of proteins detected by mass spectrometry analysis.

Gene	Protein	Present in Primary Enveloped Virus	Present in Mature Virus	Molecular Weight (kDa)	Number of Unique Peptides	Peptide Coverage (%)
**Glycoproteins**						
*UL27*	Glycoprotein B (gB)	x	√	101.1	200	63
*UL44*	Glycoprotein C (gC)	x	√	55.3	134	60
*US6*	Glycoprotein D (gD)	x	√	44.5	19	73
*US8*	Glycoprotein E (gE)	x	√	61.1	65	46
*UL22*	Glycoprotein H (gH)	x	√	88.3	82	57
*UL1*	Glycoprotein L (gL)	x	√	17.1	8	23
*US4*	Glycoprotein G (gG)	x	√	46.5	67	48
*UL10*	Glycoprotein M (gM)	x	√	45.5	6	13
*UL49.5*	Glycoprotein N (gN)	x	√	10.2	5	25
*UL53*	Glycoprotein K (gK)	x	√	35.8	5	25
*US7*	Glycoprotein I (gI)	x	√	39.9	100	45
**Envelope Proteins**						
*UL20*	pUL20	x	√	25.6	5	37
*UL34*	pUL34	x	√	27.1	55	54
*UL43*	pUL43	x	√	36.9	9	25
**Capsid Proteins**						
*UL6*	Capsid portal protein	√	√	75.1	30	86
*UL18*	Capsid triplex subunit	√	√	33.3	37	46
*UL19*	VP5 major capsid protein	√	√	105.1	106	52
**Tegument Proteins**						
*UL2*	pUL2	√	x	33.8	8	18
*UL7*	pUL7	x	√	32.5	21	63
*UL11*	pUL11	x	√	9.7	15	96
*UL14*	pUL14	x	√	23.2	31	76
*UL16*	pUL16	x	√	36.4	12	25
*UL17*	Cleavage protein	x	√	72.6	59	83
*UL21*	pUL21	×	√	60.2	47	48
*UL25*	Cleavage protein	×	√	63.1	62	63
*UL26*	Maturational protease	√	√	63.7	14	46
*UL29*	Major DNA binding protein	√	√	78.9	13	29
*UL31*	pUL31	x	√	39.5	35	72
*UL32*	pUL32	x	√	62.9	4	23
*UL36*	Egress regulating protein	√	x	327.2	79	57
*UL37*	Egress regulating protein	√	x	105.9	86	67
*UL39*	pUL39	√	√	86.1	27	58
*UL40*	pUL40	√	√	31.1	15	57
*UL41*	Host shut off protein	x	√	50.1	41	65
*UL42*	DNA processivity protein	x	√	42.6	67	63
*UL46*	VP11/12	x	√	77.6	26	38
*UL47*	VP8	√	√	80.7	47	65
*UL48*	VP16	x	√	54.1	55	58
*UL49*	VP22	√	√	26.8	148	84
*UL50*	pUL50	x	√	34.1	18	63
*UL51*	pUL51	x	√	24.9	16	86
*UL54*	bICP27	x	√	43.3	26	63
*US1.67*	pUS1.67	x	√	27.2	28	44
*US3*	Serine/threonine kinase	x	√	49.9	27	48
**Host Proteins**						
	HSP70	x	√	70.8	4	18
	Eukaryotic initiation factor 4H	x	√	30.8	26	18
	Annexin	x	√	38.9	15	75
	Alpha tubulin	x	√	50	5	23
	Beta actin	x	√	40.3	9	13
	Histone	√	√	13.9	5	29
	PCNA	√	√	28.8	11	26

√ Indicates presence, and x indicates absence.

## Data Availability

Not applicable.
